# Budd Chiari Syndrome: A Case Report on Classic Imaging Findings With Successful Intervention

**DOI:** 10.7759/cureus.43626

**Published:** 2023-08-17

**Authors:** Saurav Date, Suresh Phatak, Ashish N Ambhore, Ganesh Narwane, Deepali Trimukhe

**Affiliations:** 1 Radiodiagnosis, N. K. P. Salve Institute of Medical Sciences & Research Centre (NKPSIMS) and Lata Mangeshkar Hospital, Nagpur, IND; 2 Radiology, N. K. P. Salve Institute of Medical Sciences & Research Centre (NKPSIMS) and Lata Mangeshkar Hospital, Nagpur, IND

**Keywords:** mottled liver, inferior vena cava imaging, hepatic vein stenting, veno occlusive disease, budd chiari syndrome

## Abstract

Budd Chiari syndrome is an unusual vascular disease involving the hepatic vasculature and has significant mortality and morbidity if not treated early. Ultrasonography (USG), Doppler, computed tomography (CT), and magnetic resonance imaging (MRI) have classical imaging findings that can help make a reliable and quick diagnosis. Intervention radiology plays an important role in the treatment of these patients, helping avoid various complications and proper patient management. We are presenting a case report with classical imaging spectrum and highlighting successful intervention with hepatic vein stenting.

## Introduction

Budd Chiari syndrome is characterized by the obstruction of hepatic venous outflow anywhere between hepatic venules up to the entry of the inferior vena cava (IVC) into the right atria. Budd Chiari syndrome is an uncommon disease affecting the liver; its incidence is 0.168-4.09 per million every year, and its prevalence is 2.40-33.10 per million. If untreated, Budd Chiari syndrome leads to liver cirrhosis and has high rates of complications, including mortality. Early treatment in the form of portosystemic shunts or endovascular treatments can restore hepatic venous flow and improve liver function, thus improving prognosis. It is thus imperative that Budd Chiari syndrome be diagnosed in its early stage with accuracy thus helping avoid complications by means of early treatment [1].

## Case presentation

A 40-year-old lady presented with complaints of pain in the abdomen for one month. She also had several episodes of vomiting, which was non-projectile. On examination, there was mild tenderness in the epigastric and right hypochondriac region. The liver was palpable below the subcostal margin, suggestive of hepatomegaly.

**Figure 1:**
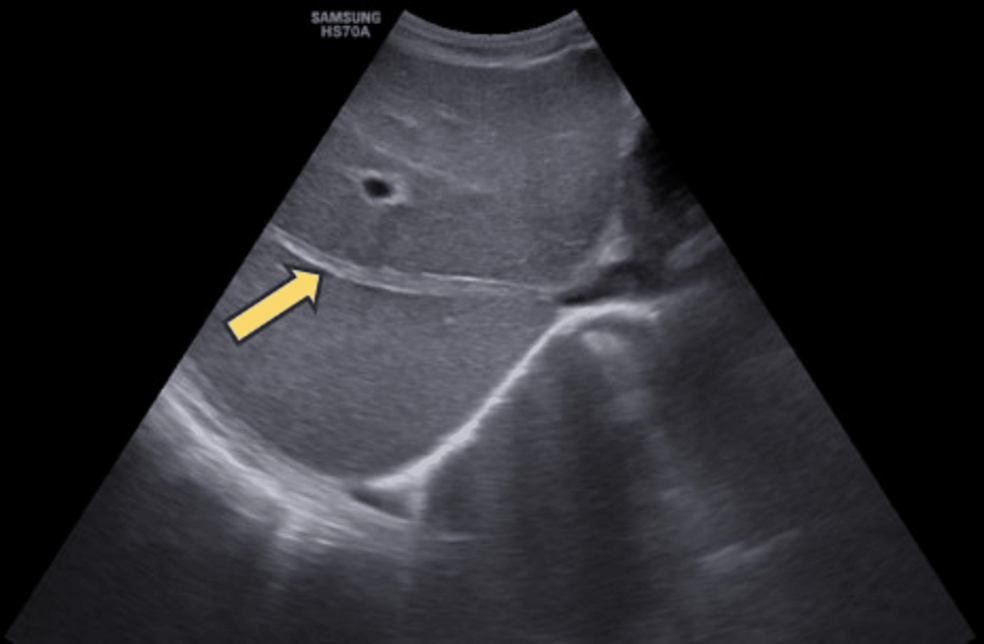
Grayscale ultrasound showing a fibrous cord replacing the middle hepatic vein (yellow arrow)

On grayscale ultrasound examination, heterogeneous echotexture was seen in the liver and a fibrous cord was noted replacing the middle hepatic vein along with the presence of mild ascites (Figure 1).

**Figure 2:**
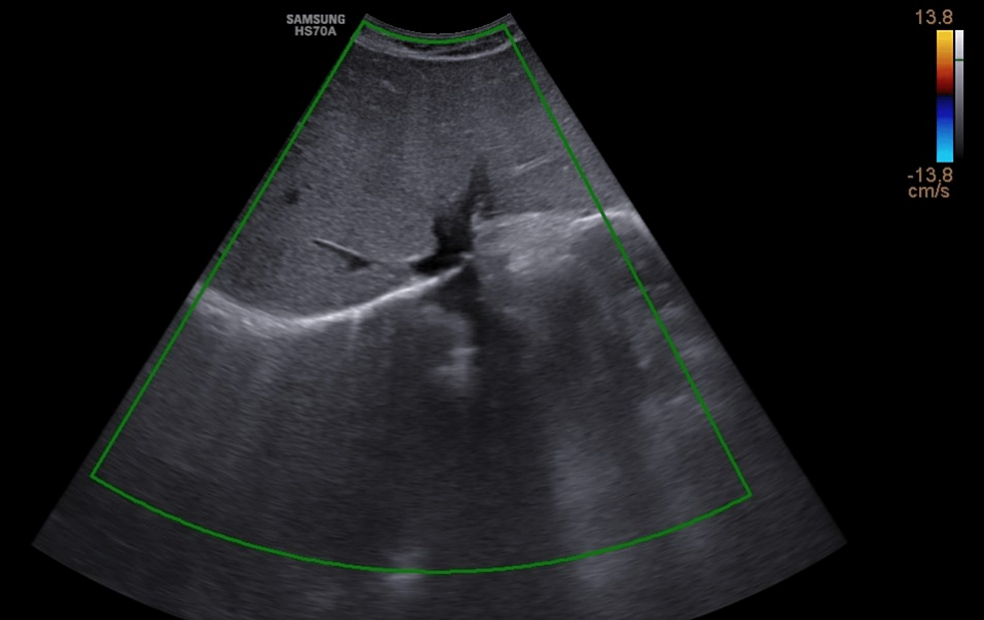
Color Doppler ultrasound showing lack of filling of the hepatic veins draining into the IVC IVC: inferior vena cava

On color Doppler, no color flow was noted in hepatic veins (Figure 2).

**Figure 3:**
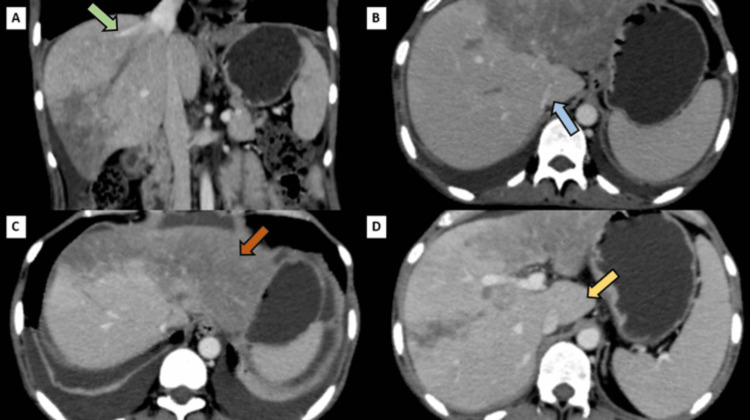
(A) Coronal reformatted CECT image showing complete non-filling of left and middle hepatic veins and partial filing of right hepatic vein(green arrow). (B) Axial section of CECT showing the slit-like intrahepatic IVC (blue arrow). (C) Axial CECT image showing the mottled appearance of the liver (orange arrow). (D) Axial section of CECT showing caudate lobe hypertrophy (yellow arrow). IVC: inferior vena cava; CECT: contrast-enhanced computed tomography

On contrast-enhanced computed tomography (CECT) scan, no contrast opacification was noted in the middle and left hepatic veins and partial opacification was noted in the right hepatic vein suggestive of complete thrombosis of middle and left hepatic veins and partial thrombosis of the right hepatic vein associated with slit-like visualization of the hepatic part of IVC. A slight enlargement of the caudate lobe was noted. The liver shows a heterogeneous mottled appearance (nutmeg liver) (Figure 3).

**Figure 4:**
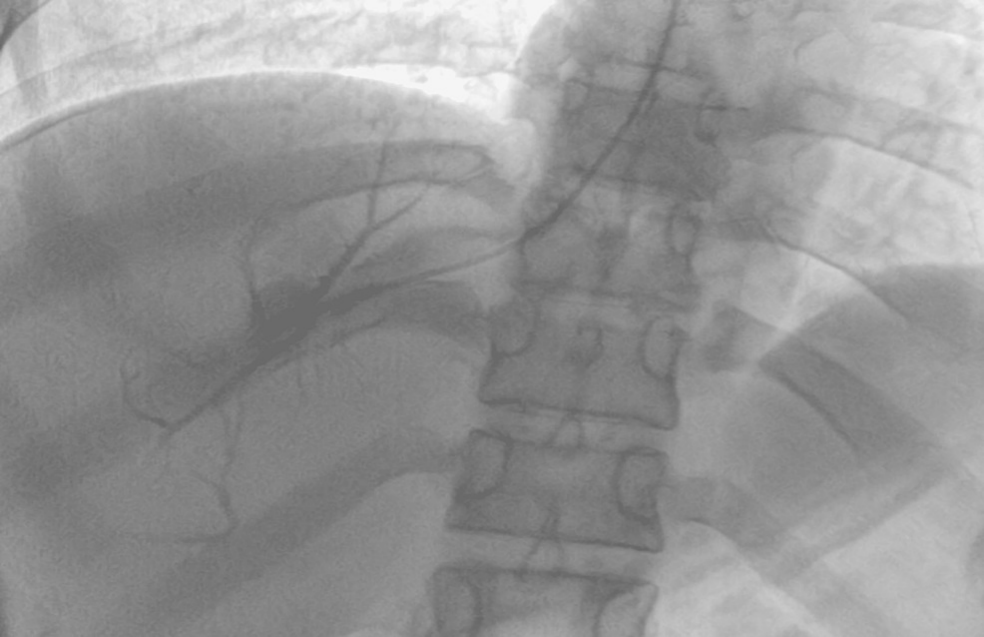
Pre-stenting venography image showing a lack of drainage of the hepatic veins into the IVC IVC: inferior vena cava

The patient underwent a diagnostic hepatic vein venography procedure, which confirmed imaging findings (Figure 4).

**Figure 5:**
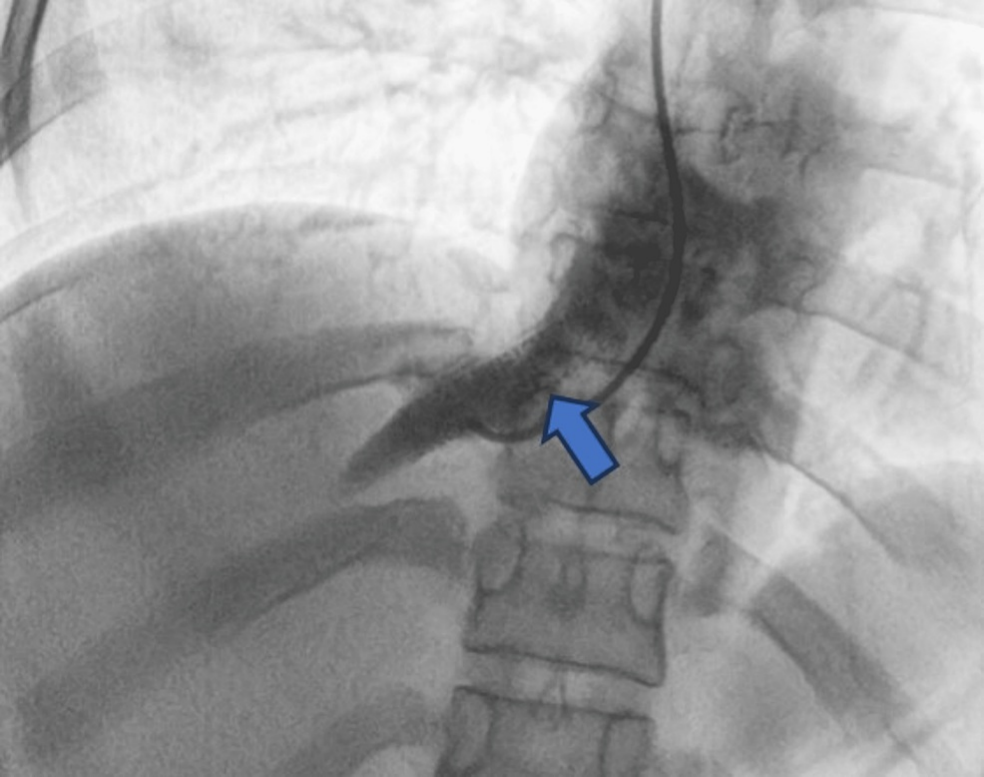
Post-stenting venography image showing the stent in-situ with well-visualized drainage into the IVC (blue arrow) IVC: inferior vena cava

Following this, hepatic vein stenting was performed successfully (Figure 5). The right internal jugular vein was used for gaining venous access. A balloon-expanding stent of size 8 x 37 mm was used for the procedure.

## Discussion

Budd-Chiari syndrome is a rare entity, which is defined by occlusion of hepatic venous outflow. Such obstruction may be at any site from hepatic venules up to the entry of the inferior vena cava into the right atria. Obstruction of at least two hepatic veins is essential, as this leads to a sufficient increase in sinusoidal pressure and reduction in sinusoidal blood flow, leading to the manifestation of clinical disease [2].

The underlying causes of Budd Chiari syndrome can be divided into primary and secondary. Thrombogenesis is the most common primary cause and patients having prethrombotic lesions and a hypercoagulable state are at risk. Secondary causes include lesions, which may compress or infiltrate the hepatic outflow tract thus causing its obstruction; for example, tumors of the liver, IVC tumors, and leiomyomas. While hepatic venous thrombosis is more common in Western countries; in Asian countries that cause mostly lies in the IVC especially in the form of membrane formation [3]. Clinical presentation of Budd Chiari syndrome ranges from asymptomatic disease to fulminant hepatic failure. The common presenting features include pain in the abdomen, abdominal distention, and dilated superficial vessels. In Western countries, the presentation is usually acute while it is usually chronic in the East. In rare cases, when all three hepatic veins are obstructed, Budd Chiari syndrome presents as fulminant hepatic failure (coagulopathy, hepatic encephalopathy, etc.) [1].

Various diagnostic modalities are useful in the diagnosis of Budd-Chiari syndrome such as conventional ultrasonography, color Doppler ultrasonography, computed tomography (CT) scans, magnetic resonance imaging (MRI), etc. On ultrasound, features such as caudate lobe hypertrophy, ascites, hepatosplenomegaly and narrowing or thrombosis of hepatic veins may be appreciated. Color Doppler studies often reveal a decreased or absence of flow in hepatic veins, reversal of hepatic vein flow, presence of intrahepatic or subcapsular collaterals, spiderweb appearance near the ostia of hepatic veins, and absent or flat hepatic wave waveform. The portal vein may show hepatofugal blood flow [1,4]. In later stages of the disease, fibrous cords may be seen replacing the hepatic veins on ultrasonography [5]. Doppler ultrasound has a sensitivity of 87.5% and a specificity of 85% [6]. Hypertrophy of the caudate lobe had a good predictive value in Budd Chiari syndrome with 100% specificity [7].

On contrast-enhanced CT imaging, thrombosis of hepatic veins can be visualized as they appear as hypoattenuating structures. Non-visualization of hepatic veins is a direct sign. Indirect signs include heterogeneous enhancement of the liver due to venous congestion, hepatomegaly, caudate lobe hypertrophy, and the presence of intrahepatic and extrahepatic collaterals [8]. In non-enhanced CT, hepatomegaly with narrowed IVC and ascites may be appreciated in acute cases. In chronic cases, caudate lobe hypertrophy and the non-visualization of IVC are predominant features [1]. In the CECT scan, the characteristic flip-flop pattern is appreciated in cases of acute Budd Chiari syndrome. It involves the early enhancement of the caudate lobe and the parts around IVC with reduced enhancement peripherally; this is followed by a decrease in central enhancement while an increase in peripheral enhancement [9]. CT venography has an 86.1% sensitivity and 97.3% specificity. CT is useful in detecting vascular abnormalities, changes in morphology, and assessment of vascular anatomy prior to endovascular interventions.

MRI is helpful, as it has the ability to detect the various aspects of Budd Chiari syndrome. The absence of signal voids along with regenerative nodules, which are iso- or hypointense on T2-weighted imaging is diagnostic. It is better than CT in the detection of regenerative nodules. In chronic cases, bright uniform or delayed peripheral retention is seen on a gadoxetate- enhanced MRI. MR angiography is useful in the detection of thrombus as well as in defining the level of venous obstruction [1]. The IVC reverse flow sign demonstrated on CT and MRI is a specific sign for IVC and mixed type of Budd Chiari syndrome, the “jet-blood” sign is peculiar to the membrane-perforated type of Budd Chiari syndrome [10].

On angiography, the spiderweb pattern of collaterals is pathognomonic of the disease; it occurs due to the presence of interconnecting collaterals. It may also depict the presence and location of a thrombus. The IVC may be seen to be compressed in the intrahepatic portion [4,11].

Without treatment, three years mortality rate is 90%. Treatment includes anticoagulation, endovascular treatments (angioplasty, stenting), transjugular intrahepatic portosystemic shunting (TIPS), and liver transplant [12]. The median survival rate at one and five years is found to be better in cases managed with endovascular recanalization treatments as compared to TIPS treatment. Also, better clinical improvement was noted in cases of recanalization [13].

## Conclusions

Budd Chiari syndrome is an infrequent disease and thus requires a high clinical index of suspicion for its diagnosis. The patient's symptoms range from none at all to acute fulminant liver failure. If untreated, it has a high incidence of mortality in patients. Early diagnosis is essential for patient survival and radiological imaging plays an important role in this pursuit.
